# An oral alpha-galactosylceramide adjuvanted *Helicobacter pylori* vaccine induces protective IL-1R- and IL-17R-dependent Th1 responses

**DOI:** 10.1038/s41541-019-0139-z

**Published:** 2019-10-25

**Authors:** Stephanie Longet, Aine Abautret-Daly, Christopher J. H. Davitt, Craig P. McEntee, Vincenzo Aversa, Monica Rosa, Ivan S. Coulter, Jan Holmgren, Sukanya Raghavan, Ed C. Lavelle

**Affiliations:** 1 Adjuvant Research Group, School of Biochemistry and Immunology, Trinity Biomedical Sciences Institute, Trinity College Dublin, Dublin 2, D02 R590 Ireland; 2 Sublimity Therapeutics Limited, Dublin City University, Alpha Innovation Campus, Old Finglas Road, Dublin, D11 KXN4 Ireland; 3 University of Gothenburg Vaccine Research Institute, Department of Microbiology and Immunology, Institute of Biomedicine, University of Gothenburg, Box 435, 405 30 Gothenburg, Sweden; 4 Centre for Research on Adaptive Nanostructures and Nanodevices & Advanced Materials Bio-Engineering Research Centre, Trinity College Dublin, Dublin 2, D02 PN40 Ireland

**Keywords:** Vaccines, Preclinical research

## Abstract

*Helicobacter pylori* causes chronic gastric infection that can lead to peptic ulcers and is an identified risk factor for gastric cancer development. Although much effort has been put into the development of a *Helicobacter pylori* vaccine over the last three decades, none has yet reached clinical application. Specific challenges pertaining to effective *H. pylori* vaccine development include the lack of proven vaccine-effective antigens and safe mucosal adjuvants to enhance local immune responses as well as the lack of accepted correlates of protection. Herein, we demonstrate that prophylactic intragastric immunisation with a whole-cell killed *H. pylori* antigen administered together with the non-toxic oral adjuvant α-galactosylceramide (α-GalCer) induced effective immune protection against *H.* pylori infection in mice, which was of similar magnitude as when using the “gold standard” cholera toxin as adjuvant. We further describe that this α-GalCer-adjuvanted vaccine formulation elicited strong intestinal and systemic Th1 responses as well as significant antigen-specific mucosal and systemic antibody responses. Finally, we report that the protective intestinal Th1 responses induced by α-GalCer are dependent on CD1d, IL-1R as well as IL-17R signalling. In summary, our results show that α-GalCer is a promising adjuvant for inclusion in an oral vaccine against *H. pylori* infection.

## Helicobacter pylori: prophylactic intragastric immunisation with oral adjuvant

Infection by *Helicobacter pylori* is highly prevalent in humans and can lead to chronic inflammation and gastric cancer, but to date no effective vaccine has been approved for clinical use owing to the lack of appropriate antigens and of safe mucosal adjuvants that can produce protective and durable immunity to the bacterium. Sukanya Raghavan, Ed Lavelle and colleagues now show that prophylactic intragastric administration of an inactivated whole-cell *H. pylori* preparation, together with the oral adjuvant α-galactosylceramide, reduced *H. pylori* infection in mice by eliciting a protective mucosal and systemic T_H_1 response. The immunisation triggered antigen-specific antibodies and interferon-γ that prevented effective colonisation of *H. pylori* after challenge in a process dependent on the CD1d, IL-1 receptor and IL-17 receptor pathways. The reported enhanced immune response to this orally adjuvanted vaccine formulation paves the way for further studies of its safety and efficacy.

## Introduction

*Helicobacter pylori* (*H. pylori*) is a gram-negative, spiral-shaped bacterium that colonises the stomach of ~50% of the worlds population^1^ and mostly causes asymptomatic chronic gastritis. However, 15–20% of infected people will develop disease related to chronic inflammation such as peptic ulcers and 1–3% of infected individuals will develop gastric cancer or mucosa-associated lymphoid tissue lymphoma.^2^

*H. pylori* bacteria are transmitted by the fecal–oral or oral–oral route. In infected individuals, the bacteria reside in the mucus of the stomach and in the duodenum. The mucosal colonisation by *H. pylori* leads to recruitment of neutrophils, monocytes, M1/M2 macrophages, mast cells, eosinophils, dendritic cells (DCs) and T and B lymphocytes to the stomach mucosa. Not surprisingly, *H. pylori*-colonised individuals have increased concentrations of pro-inflammatory cytokines such as IL-1β, IL-6, IL-8, IL-18, TNFα, IFNγ and IL-17A in the stomach.^3–6^ Th1 and Th17 responses have been found to be of prime importance for inducing protective immunity to *H. pylori* in animal models. Indeed, *H. pylori*-specific CD4^+^ T cells, mainly producing IFNγ and/or IL-17A were detected in the gastric mucosa and peripheral blood of infected individuals,^7,8^ yet these responses while apparently preventing development of manifest disease in the majority of cases, are not sufficiently effective for interrupting colonisation in most individuals. Beside antigen-specific T cells, antigen-specific IgA- and IgM-antibody-secreting cells were detected in the stomach of *H. pylori*-infected subjects.^9^ A birth cohort study in a high-endemicity area of Bangladesh demonstrated that infants fed with breast milk containing high levels of *H. pylori*-specific IgA had a significant delay in acquisition of *H. pylori* infection, suggesting that pre-existing *H.* pylori-specific IgA antibodies might help to prevent colonisation of the bacteria.^10^ The role of *H. pylori*-specific IgG in preventing colonisation of the bacteria is less clear, as immunisation of B-cell-deficient mice has shown that IgG antibodies are dispensible for protection.^11,12^ Furthermore, the transfer of serum from immunised mice did not protect recipient mice after challenge despite the presence of high levels of *H. pylori*-specific IgG in recipient mice.^13^ In humans, the frequency of *H. pylori*-specific IgG-secreting cells was shown to be higher in infected than in uninfected individuals. However, no differences in IgG titre were detected between asymptomatic and duodenal ulcers patients.^9^

The current medical management of *H. pylori* infection in patients with symptoms is based on triple therapy using long-term administration of two different antibiotics and a proton pump inhibitor.^14^ However, this therapy has several drawbacks including limited compliance, adverse reactions and risk of bacterial antibiotic resistance development. Further, even successful triple therapy leading to eradication of the current infections fails to prevent re-infections which are common in endemic settings with an average annual rate of 15–20%.^15^

Therefore, an effective *H. pylori* vaccine could have an important role both prophylactically in preventing acquisition of infection and, possibly in combination with other treatments in a therapeutic context to prevent re-infections. Since the early 1990s, vaccines based on various antigens, adjuvants and administration routes have been evaluated.^1^ A mucosal administration route, particularly the oral route, should be the most attractive for vaccination against *H. pylori* infections in order to induce an effective immune response at the site of infection.^16^ However, to date, vaccine development has been challenging owing to the complexity of the host immune response induced by *H. pylori* infection and the lack of safe mucosal adjuvants.^17^ In animal models, orally administered *H. pylori* lysate preparations, whole-cell (WC) killed bacteria or different combinations of purified *H. pylori* proteins have been evaluated as candidate vaccines. The results have uniformly shown that significant protection can be achieved provided that the specific vaccine antigens are co-administered with a potent mucosal adjuvant, whereas in the absence of such an adjuvant none of the tested vaccines have induced detectable protection.^1^

Several mucosal adjuvants have been tested together with potential *H. pylori* candidate antigens. WC or lysate preparations of *H. pylori* adjuvanted with cholera toxin (CT) or *Escherichia coli* heat-labile toxin (LT) have been found to be effective in confering protection against *H. pylori* infections.^3^ However, the toxicity of these potent enterotoxins precludes their use as mucosal vaccine adjuvants in humans. Much effort has been invested into developing non-toxic, yet adjuvant-active mutant forms of these toxins.^18,19^ Indeed, promising results have been observed in mouse models of *H. pylori* infection using candidate vaccines in combination with the detoxified mutant adjuvants like double-mutant LT and multiple-mutated CT.^19,20^ There is an urgent need to evaluate adjuvants that are potentially safe for human use and here we report promising results using the invariant natural killer T-cell (iNKT) activator, α-Galactosylceramide (α-GalCer) as an adjuvant in an inactivated WC vaccine against *H. pylori*. This adjuvant was previously found to potentiate mucosal immune responses induced by experimental *Herpes simplex* virus (HSV),^21^ enterotoxigenic *Escherichia coli*^22^ and oral cholera^23^ vaccines. The goal of the current study was to evaluate the efficiency of the α-GalCer adjuvant compared with the “gold standard” CT when orally co-administered with WC killed *H. pylori* candidate vaccine with regard to protective efficacy as well as mucosal and systemic immunogenicity. Our results demonstrate that oral immunisation with a *H. pylori* WC antigen adjuvanted with α-GalCer leads to a significant IFNγ- and CD1d-dependent reduction of bacterial loads in the stomach of *H. pylori*-infected mice, similar to the level of reduction observed when using CT as adjuvant. The vaccine-induced protection was associated with enhanced intestinal and systemic Th1 responses as well as enhanced intestinal antigen-specific IgA responses. Contrary to CT, mucosal administration of α-GalCer did not induce Th17 responses and the intestinal antigen-specific IgA responses did not require IL-17R signalling. Interestingly, we found that the induction of intestinal Th1 cellular responses by α-GalCer required IL-1R and IL-17R signalling.

Our results show that α-GalCer is an effective mucosal adjuvant for oral immunisation with an inactivated WC *H. pylori* vaccine, enhancing mucosal IgA and Th1 responses in mice and warrant evaluation of its safety and efficacy in humans.

## Results

### Oral vaccination with an α-GalCer-adjuvanted WC killed *H. pylori* vaccine induces protection against *H. pylori* infection

**Fig. 1:**
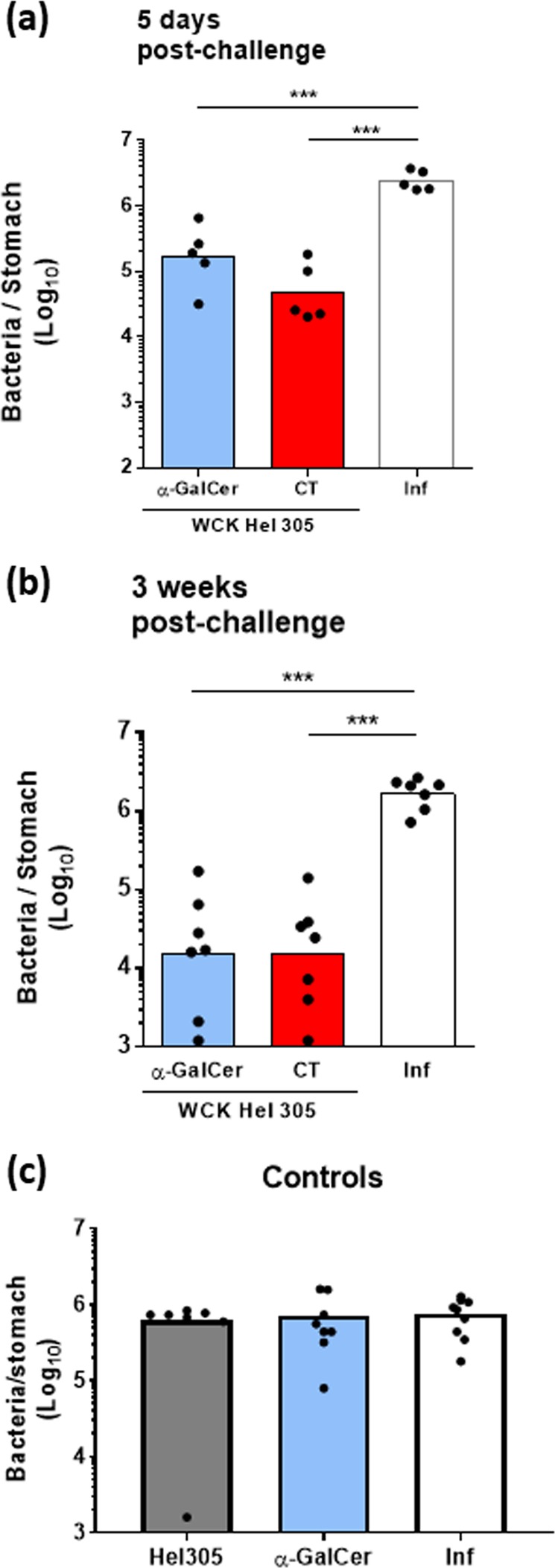
Oral vaccination with a whole-cell killed *H. pylori* antigen adjuvanted with α-GalCer leads to protective immunity against *H. pylori* challenge. WT mice were immunised intragastrically with whole-cell killed *H. pylori* Hel 305 with either α-GalCer or CT. Two weeks after the last round of immunisation, mice were infected with live *H. pylori* SS1 bacteria. Five days **a** or 3 weeks **b** post challenge, *H. pylori* colonisation was determined by quantitative culture. As controls, WT mice were immunised intragastrically with either whole-cell killed *H. pylori* Hel 305 or α-GalCer alone and *H. pylori* colonisation was determined 3 weeks post challenge **c**. Unimmunised mice challenged at the same time-point served as infection controls (inf). All data are expressed as Log10 values of bacteria per stomach and are representative of three independent experiments. Data represent geometric means. All experiments were performed with 5–7 mice per group. ***p < 0.001

Identifying safe and orally active adjuvants that drive protective mucosal and systemic *H. pylori*-specific immune responses is essential for the development of an effective vaccine. In order to determine the potential of the oral adjuvant α-GalCer, mice were immunised with a WC *H. pylori* antigen and α-GalCer or the gold standard oral adjuvant CT for comparison. Two weeks after the last of two rounds of immunisation, mice were challenged with live *H. pylori*. The effect of immunisation on both acute and chronic infection was determined by measuring the bacterial load in the stomach 5 days or 3 weeks after challenge by quantitative culture and compared with the bacterial load in unimmunised mice (infection control group). We have previously reported that repeated intragastric administration of WC *H. pylori* antigen alone or CT adjuvant alone does not confer any protection against *H. pylori* infection.^20^ Oral immunisation with WC *H. pylori* antigen adjuvanted with α-GalCer led to a significant reduction of bacterial loads in stomach tissues compared with the infection control group (Supplementary Fig. 1a), comparable to the levels of reduction seen in mice immunised with WC *H. pylori* antigen adjuvanted with CT. Specifically, 5 days and 3 weeks post challenge, there was a 20-fold (Fig. 1a) and 100-fold reduction (Fig. 1b), respectively, observed in mice immunised with the α-GalCer containing vaccine compared with the infection control groups. We also demonstrated that the combination of WC *H. pylori* antigen and adjuvant was needed for detectable immune protection; thus, immunisation with WC *H. pylori* or α-GalCer alone gave no protection against challenge (Fig. 1c). Strikingly, a significant increase in IFNγ gene expression was measured in gastric tissue from mice vaccinated with WC *H. pylori* antigen and α-GalCer compared with the infection control group (Supplementary Fig. 1a), whereas no significant increase in IL-17A gene expression was observed 3 weeks post infection (Supplementary Fig. 1b). IFNγ gene expression following vaccination with antigen and α-GalCer was comparable to that induced by the CT containing vaccine (Supplementary Fig. 1a). In addition to these local responses, serum antigen-specific IgG titres (Supplementary Fig. 2a) and splenocyte proliferation after stimulation were enhanced (Supplementary Fig. 2b and 2c) in mice orally vaccinated with the α-GalCer-adjuvanted formulation compared with the infection control group, 3 weeks after challenge. Given that the *H. pylori*-specific IgG antibodies are not protective during immunisation,^11^ the measurement of high IgG titre is only indicative of successful immunisation.

### Oral immunisation with WC killed *H. pylori* adjuvanted with α-GalCer induces systemic and mucosal Th1 responses

**Fig. 2:**
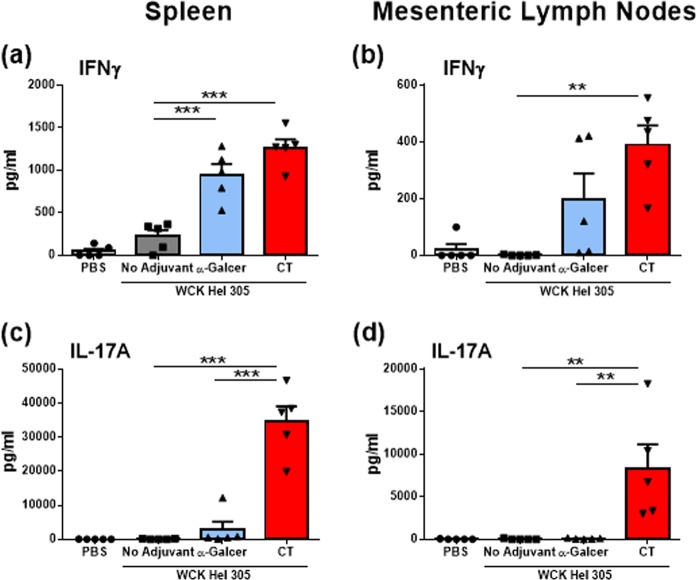
Oral vaccination with Hel 305 and α-GalCer selectively promotes *H. pylori* antigen-specific Th1 responses. Mice were immunised intragastrically with whole-cell killed *H. pylori* Hel 305 with or without either α-GalCer or CT. Two weeks after the last round of immunisation, cells were isolated from the spleen **a**, **c** and mesenteric lymph nodes **b**, **d** and restimulated ex vivo with purified MP305 for 72 hours. Supernatants were harvested and analysed for either IFNγ **a**, **b** or IL-17A **c**, **d** by ELISA. Groups of mice administered with Phosphate-Buffered Saline (PBS) are shown as negative controls. Results represent cytokine concentrations (mean + Standard Error of the Mean (SEM)) for five mice per group. For all groups of mice, the cytokine levels without antigen stimulation was substracted from the levels after stimulation. Data representative of three independent experiments with *n* = 5 mice/group and experiment. **p < 0.01, *** < 0.001

T-cell responses and particularly Th1 and Th17 cells have previously been shown to play a crucial role in vaccine-induced protection against *H. pylori* infection using CT or other enterotoxin-adjuvanted formulations.^19,24^ The observed increase of IFNγ gene expression in stomach tissues following immunisation with WC *H. pylori* antigen and α-GalCer and challenge with *H. pylori* suggests the induction of a local Th1 response. To assess vaccine-induced antigen-specific Th1 and Th17 responses, mice were immunised intragastrically in two rounds with WC *H. pylori* antigen alone or antigen adjuvanted with α-GalCer or CT, and cellular responses in spleens and mesenteric lymph nodes (MLN) were analysed. Two weeks after the last immunisation, isolated splenocytes and MLN cells were restimulated in vitro with *H. pylori* strain 305 membrane protein (MP305). Significantly, higher levels of IFNγ were found in supernatants from restimulated splenocytes of mice vaccinated with WC *H. pylori* antigen and α-GalCer compared with splenocytes from mice immunised with the antigen alone. CT-adjuvanted WC antigen induced comparable responses to the α-GalCer-adjuvanted vaccine in promoting splenocyte Th1 responses (Fig. 2a). Vaccination with antigen and α-GalCer or CT also resulted in enhanced MP305-specific Th1 responses in MLNs compared with immunisation with antigen alone (Fig. 2b). However, in contrast to CT, which induced strong Th17 responses in addition to the IFNγ responses, the α-GalCer-adjuvanted formulation did not promote *H. pylori* antigen-specific Th17 responses in either spleen (Fig. 2c) or MLNs (Fig. 2d). In addition to enhancing Th1 responses, immunisation with WC *H. pylori* antigen and α-GalCer promoted an increase in antigen-specific spleen and MLN cell proliferation after stimulation (Supplementary Fig. 3a and 3b). In summary, potent activation of iNKT cells by α-GalCer leads to the selective enhancement of antigen-specific Th1 responses against *H. pylori* infection in mice.

### Oral administration of α-GalCer enhances intestinal antigen-specific IgA responses to a WC killed *H. pylori* antigen

**Fig. 3:**
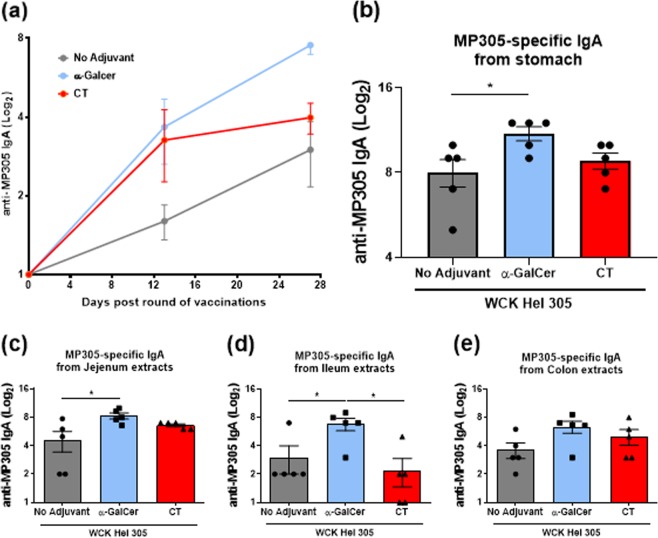
α-GalCer enhances *H. pylori* antigen-specific intestinal IgA responses. Mice were intragastrically immunised with whole-cell killed *H. pylori* Hel 305 with or without either α-GalCer or CT on days 0 and 1 then, on days 14 and 15. MP305-specific IgA titres in fecal pellet supernatants were determined by ELISA before each round of immunisation and 2 weeks after the last round of immunisation. The graph line shows the evolution of IgA titres post immunisation for each immunised group of mice **a**. Two weeks after the second round of immunisation, mice were perfused to remove the blood from the organs and tissue was collected. *H. pylori* MP305-specific IgA titres were measured in stomach **b** jejunum **c**, ileum **d**, and colon **e** extracts. Results represent antibody titres, Log2 (mean + SEM). Data representative of three independent experiments with *n* = 5 mice/group and experiment. *p < 0.05, **p < 0.01, ***p < 0.001

We next determined intestinal *H. pylori*-specific IgA responses after two rounds of oral immunisation with WC *H. pylori* antigen alone or adjuvanted with α-GalCer or CT. Oral co-administration of the antigen and α-GalCer enhanced MP305-specific IgA responses in fecal pellet supernatants after two immunisation rounds (Fig. 3a) compared with the limited responses seen in mice vaccinated with the *H. pylori* antigen alone. Notably, antigen-specific fecal IgA responses induced by antigen and α-GalCer after the second round of immunisation were even higher than those generated by vaccination with the antigen and CT (Fig. 3a). Further, by analysing antigen-specific IgA production from the stomach tissue and different segments of the intestine using the Perfext method,^25^ it was also observed that vaccination with the α-GalCer-adjuvanted WC *H. pylori* antigen significantly enhanced antigen-specific IgA responses in the stomach (Fig. 3b), the jejunum (Fig. 3c) and ileum (Fig. 3d) tissues compared to the WC *H. pylori* antigen alone and to a lesser extent in the colon (Fig. 3e). Similar to the fecal antibody titres, a significantly higher antigen-specific IgA response was observed following oral vaccination with WC *H. pylori* antigen adjuvanted with α-GalCer compared with the CT-adjuvanted group in ileal extracts (Fig. 3d). Given that Th17 cells have been shown to be crucial for mucosal antigen-specific IgA responses,^26^ we next asked whether the enhanced MP305-specific IgA responses induced by the α-GalCer or CT-adjuvanted *H. pylori* vaccine were dependent on intact IL-17R signalling. Thus, WT and IL-17R^−/−^ mice were immunised with antigen alone or antigen adjuvanted with α-GalCer or CT. Contrary to the findings with CT, it was observed that the lack of IL-17R did not abrogate the ability of vaccination with antigen and α-GalCer to enhance MP305-specific IgA responses in either fecal pellet extracts (Supplementary Fig. 4a) or in jejunal (Supplementary Fig. 4b), ileal (Supplementary Fig. 4c) or colonic tissue extracts (Supplementary Fig. 4d). These findings show that IL-17R signalling is dispensable for the induction of antigen-specific IgA responses following vaccination with antigen and α-GalCer. This is also consistent with the lack of detectable Th17 responses after immunisation with the α-GalCer-adjuvanted *H. pylori* vaccine.

### Induction of Th1 responses by α-GalCer is dependent on intact IL-17R and IL-1R signalling

**Fig. 4:**
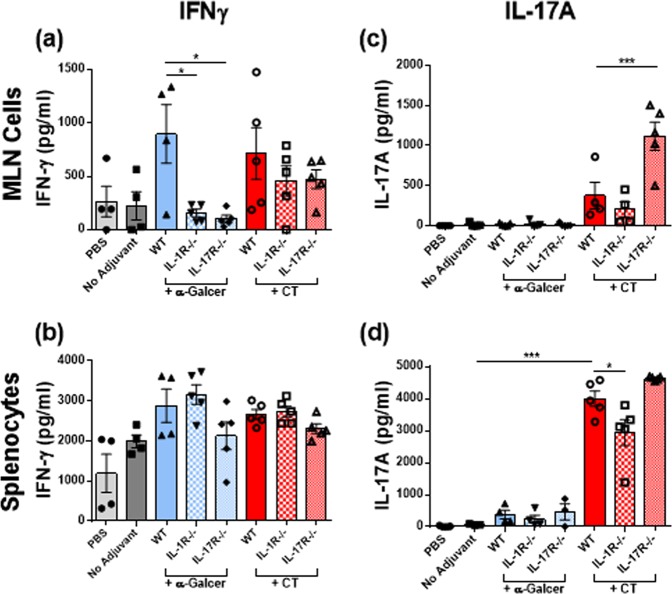
Induction of intestinal but not systemic Th1-type responses by α-GalCer requires IL-1R and IL-17R. WT, IL-17R^−/−^ and IL-1R^−/−^ mice were immunised intragastrically with whole-cell killed *H. pylori* Hel 305 with or without either α-GalCer or CT. Two weeks after the last round of immunisation, cells were isolated from the spleen and mesenteric lymph nodes (MLN) and restimulated ex vivo with purified MP305 for 72 hours. Supernatants were harvested and analysed for IFNγ **a**, **b** and IL-17A **c**, **d** by ELISA. Results present cytokine concentrations (mean + SEM) for five mice per group. For all groups of mice, the cytokine levels without antigen stimulation was substracted from the levels after stimulation. Data representative of two independent experiments with *n* = 5 mice/group and experiment. *p < 0.05, **p < 0.01, ***p < 0.001

It has been shown that IL-17R and IL-1R signalling influenced mucosal Th1 responses in a *Chlamydia muridarum* pulmonary infection model^27^ and in a *Candida albicans* mucosal infection model^28^ as well as in chronic inflammation.^29^ Here, we addressed whether IL-17R and/or IL-1R signalling was required for systemic and local α-GalCer-induced Th1 responses to *H. pylori* antigens. WT, IL-17R^−/−^ and IL-1R^−/−^ mice were immunised with *H. pylori* antigen given alone or adjuvanted with α-GalCer or CT. MLN cells and splenocytes were restimulated in vitro with *H. pylori* MP305 and IFNγ and IL-17A secretion was assessed in the supernatants. As before, oral immunisation with *H. pylori* antigen adjuvanted with α-GalCer enhanced Th1 responses in the MLN and spleen of WT mice compared to vaccination with antigen alone (Fig. 4a, b). However, in vitro restimulated MLN cells from IL-1R^−/−^ and IL-17R^−/−^ mice vaccinated with WC *H. pylori* antigen and α-GalCer produced significantly lower concentrations of IFNγ compared with MLN cells from similarly immunised WT mice (Fig. 4a), whereas no significant differences in IFNγ production were observed from stimulated splenocytes of WT, IL-1R^−/−^ and IL-17R^−/−^ mice (Fig. 4b). In contrast, the enhancement in MLN and splenic antigen-specific Th1 responses by CT was intact in WT as well as IL-1R^−/−^ or IL-17R^−/−^ mice (Fig. 4a, b). As previously shown in this study, oral vaccination with α-GalCer did not promote Th17 responses in splenocytes or MLNs from WT and here we also show that the adjuvant did not enhance Th17 responses in the spleen or MLN of IL-17R^−/−^ or IL-1R^−/−^ mice (Fig. 4c, d), whereas the CT-adjuvanted vaccine effectively promoted a Th17 response in WT mice (Fig. 4c, d). Restimulated splenocytes from IL-1R^−/−^ mice vaccinated with the CT-adjuvanted WC *H. pylori* antigen secreted significantly lower concentrations of IL-17A compared with WT mice (Fig. 4d). In summary, α-GalCer is a potent inducer of mucosal Th1 responses whose induction is dependent on IL-17R as well as IL-1R signalling.

### IFNγ is essential for protective immunity induced by oral vaccination with WC *H. pylori* antigen adjuvanted with α-GalCer

**Fig. 5:**
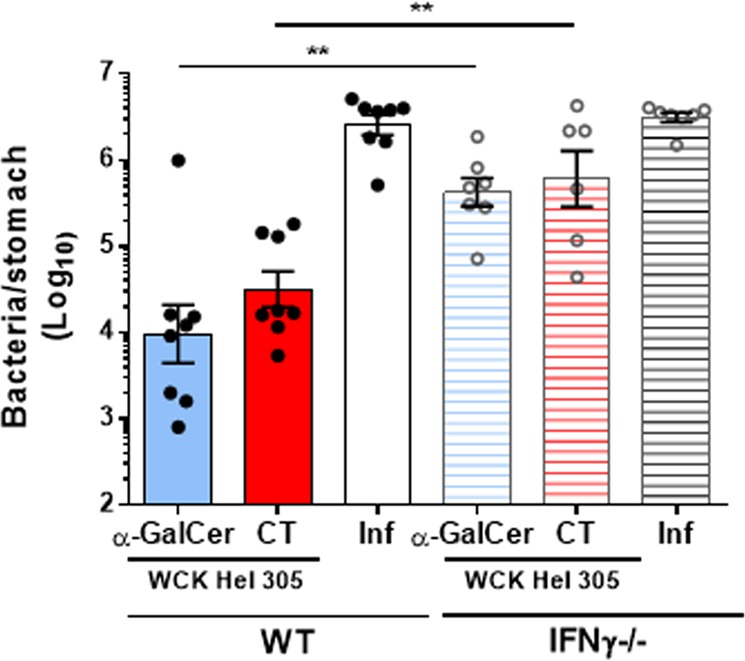
The induction of protective immunity by oral vaccination with whole-cell killed *H. pylori* adjuvanted with α-GalCer requires IFNγ. WT and IFNγ^−/−^ mice were immunised intragastrically with whole-cell killed *H. pylori* Hel 305 with either α-GalCer or CT. Two weeks after the last round of immunisation, mice were challenged with live *H. pylori* SS1 bacteria. Three weeks post challenge, *H. pylori* colonisation was determined by quantitative culture. Unimmunised mice challenged at the same time-point served as infection controls (inf). Data represent geometric means + SEM. The experiment was performed with 6–8 mice per group. **p < 0.01

Having shown that oral delivery of α-GalCer significantly enhanced immune protection induced by a WC *H. pylori* antigen and promoted both local (MLN) and systemic (splenic) Th1 responses, we evaluated the importance of the IFNγ response for α-GalCer driven protection. Challenge studies were performed in WT and IFNγ^−/−^ mice. *H. pylori* antigen-specific IgA responses in intestinal tissue extracts and IgG responses in serum as well as splenocyte T-cell proliferation were compared between the WT and IFNγ^−/−^ mice. No significant differences in intestinal IgA (Supplementary Fig. 5a) as well as systemic IgG and IgG1 antibody responses (Supplementary Fig. 5b and 5c) or antigen-specific splenocyte proliferation after stimulation (Supplementary Fig. 5e and 5f) were observed between WT and IFNγ^−/−^ mice immunised with either the α-GalCer- or CT-adjuvanted *H. pylori* antigen formulations. A reduction in *H. pylori*-specific IgG2c was seen in the IFNγ^−/−^ mice immunised with either the α-GalCer- or CT-adjuvanted *H. pylori* antigen formulations (Supplementary Fig. 5d). In contrast, there was a marked difference with regard to induction of protection against challenge. Although WT mice vaccinated with antigen and α-GalCer showed a significant reduction of bacteria in the stomach compared with unimmunised mice (infection control group) (Fig. 5), in the IFNγ^−/−^ mice no significant reduction in bacterial levels could be seen (Fig. 5). The dependency on IFNγ for vaccine-induced protection against *H. pylori* was also observed in WT and IFNγ^−/−^ mice vaccinated with WC *H. pylori* antigen and CT (Fig. 5).

### Protective immune responses induced by oral vaccination with α-GalCer-adjuvanted WC *H. pylori* antigen are dependent on CD1d

**Fig. 6:**
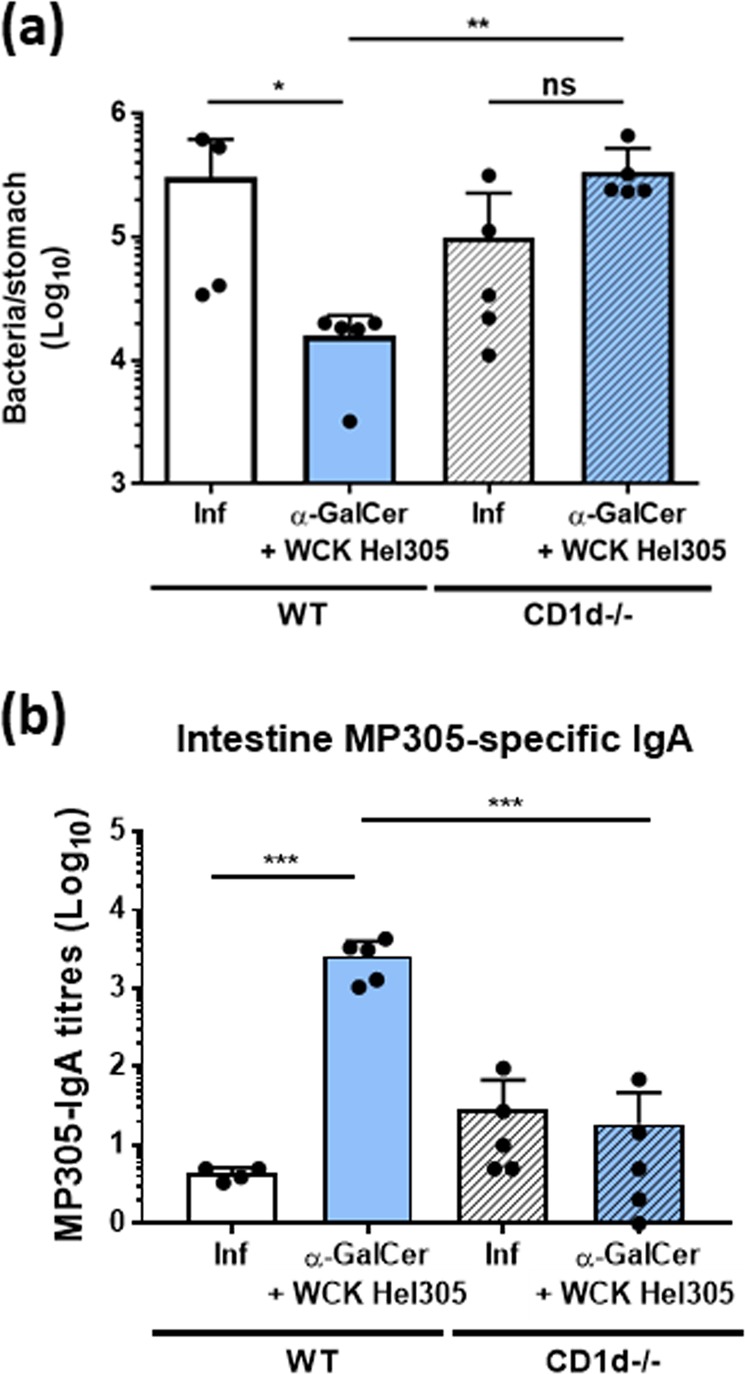
Protective immunity and antigen-specific IgA responses induced by oral vaccination with whole-cell killed *H. pylori* adjuvanted with α-GalCer requires the antigen-presenting protein CD1d. WT and CD1d^−/−^ mice were immunised intragastrically with whole-cell killed *H. pylori* Hel 305 with α-GalCer. Two weeks after the last round of immunisation, mice were challenged with live *H. pylori* SS1 bacteria. Three weeks post challenge, *H. pylori* colonisation was determined by quantitative culture **a** and intestinal tissue was collected. MP305-IgA titres in intestinal tissues were determined by ELISA **b**. Unimmunised mice challenged at the same time-point served as infection controls (inf). Data represent geometric means + SEM. The experiment was performed with 4–5 mice per group. *p < 0.05, **p < 0.01, ***p < 0.001

It is known that α-GalCer is presented by the antigen-presenting protein CD1d expressed on immune cells including DC and B cells^30^ and on intestinal epithelial cells^31^ leading to iNKT cell activation, which subsequently promotes DC and B-cell maturation through cytokine release. In order to evaluate whether protection of vaccinated mice with the α-GalCer-adjuvanted *H. pylori* antigen formulation required the CD1d pathway for α-GalCer presentation, challenge studies were performed in WT and CD1d^−/−^ mice. As observed in the previous challenge studies, WT mice immunised with *H. pylori* antigen and α-GalCer showed a significant reduction of bacteria in the stomach compared with unimmunised mice (infection control group) (Fig. 6a). However, in CD1d^−/−^ mice, no significant reduction in bacterial numbers in the stomach could be observed (Fig. 6a). Furthermore, oral immunisation of CD1d^−/−^ mice with WC *H. pylori* antigen adjuvanted with α-GalCer did not induce a significant antigen-specific IgA response in intestinal tissues (Fig. 6b) or serum IgG response (Supplementary Fig. 6) compared with WT mice similarly vaccinated. In summary, CD1d is essential for the potential of oral vaccination with WC *H. pylori* antigen and α-GalCer to induce protective immunity.

## Discussion

Although significant resources have been invested over recent decades into the development of a *H. pylori* vaccine, no such vaccine has yet reached clinical application. The induction of protective immunity in humans remains challenging and identifying safe yet potent mucosal adjuvants to enhance local protective immune responses remains a critical roadblock for the development of an effective *H. pylori* vaccine.^1,3^

The medical potential of iNKT cell activator α-GalCer was first recognised for its anti-tumour properties.^32,33^ More recently, its ability to enhance local immune responses following mucosal administration has been described in animal models in the context of vaccination against HIV^34^ influenza^35^ and HSV-2.^21^ In addition, α-GalCer has been tested in clinical trials to stimulate iNKT cells in the context of solid tumours^36^ or hepatitis B infections^37^ and its safety has been confirmed after systemic administration, which indicates that it could be a promising mucosal adjuvant for humans.^36,37^ In the current study, we observed that intragastric administration of WC *H. pylori* antigen and α-GalCer was as effective as administration of the antigen adjuvanted with CT for promoting protective immune responses against *H. pylori* infection. We demonstrated that α-GalCer enhanced the protective efficacy of this WC killed *H. pylori* mucosal candidate antigen by inducing strong intestinal and systemic Th1 responses but not Th17 responses to *H. pylori*, contrary to CT that promoted both Th17 and Th1 responses.

α-GalCer was also effective in eliciting intestinal antigen-specific IgA responses, even surpassing CT in the induction of antigen-specific IgA responses in fecal pellet extracts and ileal tissues. Hirota et al.^26^ showed that local Th17 cells were essential to generate intestinal T-cell-dependent IgA production. In addition, Cao and colleagues^38^ reported that IL-17A induced pIgR expression on the intestinal epithelium and enhanced transepithelial IgA secretion. It was also demonstrated that IL-17 was required for the induction of cell-mediated immunity and antigen-specific serum and mucosal antibody responses following oral immunisation with ovalbumin and CT.^39^ Our present data confirmed the association between antigen-specific IgA responses enhanced by CT and IL-17R signalling. However, contrary to our findings with CT, we observed that IL-17R signalling was not required for the ability of α-GalCer to enhance antigen-specific IgA responses in fecal pellets and intestinal tissue extracts. Our results suggest that α-GalCer and CT use two distinct pathways for enhancing antigen-specific intestinal IgA and demonstrate that in certain contexts, IL-17 is dispensable for IgA transport across epithelial surfaces.

Addressing the mechanisms by which vaccines induce protective immunity is essential in vaccine regulatory processes to identify immunological assays that could serve as surrogates of protection^40^ and for resolving mechanistic pathways that may serve as targets for development of improved vaccination approaches. The involvement of the IL-17 receptor in the induction of Th1 response has been demonstrated in models of *Chlamydia muridarum* pulmonary infection^27^ and *Candida albicans* oral infection,^28^ where reduced mucosal Th1 responses were observed in IL-17RA^−/−^ mice. The role for IL-1 in promoting Th1 responses is less clear but has been reported in the context of autoimmunity.^29^ Indeed, excessive IL-1 signalling may indirectly lead to enhanced Th1 responses.^29^ In order to further dissect the mechanisms of action of α-GalCer in comparison with CT in the induction of Th1 responses, we analysed the requirement for IL-17R and IL-1R signalling after immunisation with WC *H. pylori* antigen adjuvanted with α-GalCer or CT. Here we found that intestinal Th1 responses induced by the WC *H. pylori* antigen and α-GalCer required both IL-1R and IL-17R signalling. Strikingly, the requirement for this signalling pathway was only observed at the level of MLNs and was specific to α-GalCer, as it was not observed when using CT as an adjuvant. These data suggest different mechanisms underlying protective Th1 immune responses generated by α-GalCer in comparison with CT. An important role for IFNγ and IL-17 in protection against *H. pylori* infection after oral immunisation with *H. pylori* antigens and CT has been previously reported.^24,41^ Here, we demonstrate for the first time that protection from *H. pylori* infection elicited by WC *H. pylori* antigen and α-GalCer was fully dependent on IFNγ. Finally, we confirmed that the presentation of α-GalCer by the antigen-presenting protein CD1d was crucial to induce protective immune responses.

**Fig. 7:**
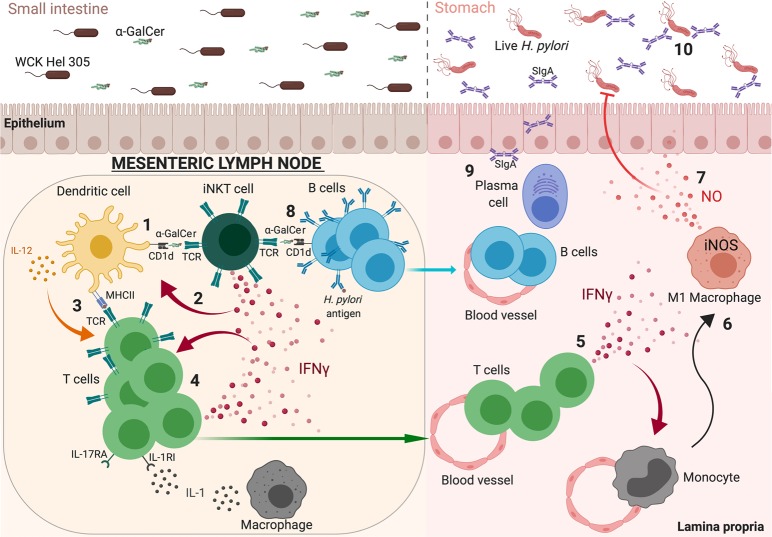
Schematic cartoon summarising the potential mechanisms underlying the efficacy of α-GalCer as a mucosal adjuvant in a whole-cell *H. pylori* vaccine. 1) The iNKT cell activator α-GalCer is presented by CD1d expressed on cells including DCs, which have taken up *H. pylori* antigens, to iNKT cells in mesenteric lymph nodes. 2) Activated iNKT cells secrete IFNγ that promotes DC maturation. 3) The IFNγ-matured DCs in their turn present processed *H. pylori* antigens on MHCII and secrete IL-12. 4) *H. pylori* peptide presentation and IL-12 secretion activate naive CD4^+^ T cells to become IFNγ-secreting antigen-specific Th1 cells, which expand locally in the presence of IFNγ by a positive feedback loop mechanism before exiting via the draining lymph into the blood stream. 5) After a *H. pylori* infection, these circulating intestine-derived CD4^+^ Th1 cells can then migrate into the stomach mucosa and secrete IFNγ. 6) This migration polarises the recruited inflammatory monocytes to the M1 macrophage phenotype. 7) M1 macrophages express iNOS and secrete NO, which leads to a reduction of *H. pylori* colonisation in the stomach. A specific requirement for IL-1R and IL-17R signalling in intestinal CD4^+^ T-cell IFNγ responses needs to be further elucidated. We speculate that IL-1 could be produced and secreted by macrophages. 8) Activated iNKT cells can also directly stimulate B cells to promote plasma cell differentiation. 9) This leads to an enhancement of SIgA in the stomach. 10) SIgA might also play a role in blocking infection. IFNγ: interferon γ; IL-1RI: interleukin 1 receptor I; IL-12: interleukin 12; IL-17RA: interleukin 17 receptor A; iNKT cell: invariant natural killer T cells; iNOS: inducible nitric oxide synthase; MHCII: major histocompatibility complex II; NO: nitric oxide; SIgA: secretory immunoglobulin A; TCR: T-cell receptor; WCK: Whole-cell killed. This Figure used images that were created with Biorender.com are permissible to use

These data indicate that orally administered α-GalCer is presented by CD1d to iNKT cells, which results in the activation of DCs,^30^ which selectively promote Th1 responses. The adjuvant also strongly promotes antigen-specific antibody responses. The elevated levels of gastric IFNγ induced in vaccinated mice by the α-GalCer-adjuvanted *H. pylori* antigen may generate a local pro-inflammatory environment, which can activate M1 macrophages and protect mice from *H. pylori* infection. An accumulation of inflammatory monocytes and macrophages has been previously shown in the stomach of mucosally vaccinated mice post infection^42^ (Fig. 7). The specific requirement for IL-1R and IL-17R signalling in intestinal CD4^+^ T-cell IFNγ responses is noteworthy and needs to be further elucidated.

In summary, we demonstrate that the oral adjuvant α-GalCer induces protective mucosal Th1 responses when co-administered with *H. pylori* WC candidate antigen through IL-1 and IL-17R signalling.

## Methods

### Ethics statement

Animals were maintained according to the regulations of the EU and the Irish Department of Health and all procedures performed were conducted under animal license number B100/3321 and were approved by the Trinity College Dublin Animal Research Ethics Committee (Ethical Approval Number 091210). Animal care and all protocols adhered to the regulations and guidelines established by the Health Products Regulatory Authority which is the competent authority in Ireland responsible for the implementation of EU legislation (Directive 2010/63/EU) for the protection of animals used for scientific purposes.

### Animals

For the immunisation experiments, specific-pathogen-free female C57BL/6, IL-17RA^−/−^ and IL-1RI^−/−^ mice were obtained from Charles River Laboratories, Inc., and were used at 12–16 weeks of age. Animals were maintained according to the regulations of the EU and the Irish Department of Health and all procedures performed were conducted under animal license number B100/3321 and were approved by the Trinity College Dublin Animal Research Ethics Committee (Ethical Approval Number 091210). For the challenge experiments, 6–8-week old, specific-pathogen-free female C57BL/6 mice were purchased from Taconic (Ejby, Denmark). Age-matched IFN-γ-deficient (IFN-γ^−/−^) mice on a C57BL/6 background^43^ and CD1d-deficient (CD1d^−/−^) mice on a C57BL/6 background were bred at the Laboratory for Experimental Biomedicine, University of Gothenburg, and all mice were housed in microisolators for the duration of the study. All experiments were approved by the local ethics committee for animal experiments (Gothenburg, Sweden; ethical approval number 189/14).

### *H. pylori* strains and antigens

Mice were immunised with a *H. pylori* WC antigen prepared from a clinical isolate selected from the *H. pylori* strain collection (University of Gothenburg, Sweden), designated Hel 305. The *H. pylori* Hel 305 strain was grown on plates and subsequently inactivated with formalin as previously described.^44^ The *H. pylori* mouse-adapted strain SS1^45^ was used for all infections. Both Hel 305 and *H. pylori* SS1 are CagA^+^ and VacA^+^.^46^ The lysates and whole-membrane proteins from Hel 305 were prepared as previously detailed in Ghiara et al.^47^ and in Lindholm et al.,^48^ respectively.

### Immunisation, *H. pylori* challenge and bacterial quantification in the stomach

Groups of mice (*n* = 5) were immunised orally four times in two rounds on days 0 and 1; 14 and 15. One hour prior to immunisation, food was withdrawn. Mice were orally gavaged with 200 µl of 0.3 m sodium bicarbonate buffer. After 20 min, mice were gavaged with 200 µl of sterile Phosphate-buffered saline (PBS) containing 1 × 10^9^ formalin-killed *H. pylori* Hel 305 with or without α-GalCer (10 μg) (Avanti Lipids) or CT (10 μg) (LIST Biologicals), or with PBS alone. Antibody and cellular responses were analysed on day 28. For the challenge experiments, groups of mice (*n* = 7–8) were immunised orally six times in three rounds on days 0 and 1; 14 and 15; 28 and 29. Mice were immunised with 300 µl of 3% sodium carbonate buffer containing 1 × 10^9^ formalin-killed *H. pylori* Hel 305 with or without α-GalCer (20 μg) (Avanti Lipids) or CT (7.5 μg) (Sigma Aldrich, St. Louis, MO), or with PBS alone. The adjuvant α-GalCer was dissolved in a solution containing oils and surfactants from Sublimity Therapeutics Ltd. Two weeks after the last immunisation, all mice were orally challenged with 3 × 10^8^ live *H. pylori* SS1 in brucella broth (OD_600_ adjusted to 1.5) using a feeding needle under anaesthesia.^49^ Animals were killed 5 days or 3 weeks post challenge and the number of bacteria in the stomach were determined by quantitative culture as previously detailed in Raghavan et al.^44^

### Analysis of cellular immune responses and splenocyte proliferation

The killing of mice and subsequent analysis of cellular responses were performed as previously described.^23^ Splenocytes and MLN cells were plated at 1 × 10^6^ cells/ml in complete Roswell Park Memorial Institute (cRPMI). Cells were stimulated for 72 h at 37 °C/5%CO_2_ with the following stimuli: boiled Hel 305 lysate antigens (1 µg/ml) or purified MP305 (1 µg/ml). Seventy-two hours post stimulation, IFNγ and IL-17A were measured in collected supernatants using the Mouse IFNγ and IL-17A DuoSet ELISA kits (R&D systems). For splenocyte proliferation, the cells were pulsed with 1 µCi of [^3^H] Thymidine (Amersham Bioscience Buckinghamshire, UK) for 7 h. The cellular DNA was collected with a cell harvester (Skatron) on glass fibre filters (Wallac) and assayed for ^3^H incorporation using a liquid scintillation counter (Beckman, LKB, Bromma, Sweden).

### Collection of fecal and serum samples

Fecal pellet supernatants and serum samples were collected and prepared as previously described.^22,23^ Mice were placed into individual cages and five fresh fecal pellets were collected in 500 µl of cold fecal pellet buffer (0.1 mg/ml Soybean trypsin inhibitor (Sigma Aldrich), 1% Bovine Serum Albumin (BSA), 25 mM ethylenediaminetetraacetic acid (EDTA) (Gibco), 1 mM PEFABloc (Sigma Aldrich), 50% Glycerol in 1× PBS)) and kept on ice for 4 h. Samples were then weighed, emulsified, centrifuged at 15,400 × *g* for 5 min at 4 °C and supernatants were stored at −20 °C until further use. Blood was obtained from mice by incision into the tail vein. Samples were left to coagulate overnight at 4 °C and then centrifuged at 9200 × *g* for 10 mins. The serum was stored at −20 °C until further use.

### Preparation of intestinal tissues

IgA responses in stomach, jejenum, ileum and colon were collected using the Perfusion-Extraction (PERFEXT) method as previously described.^22,25,50^ After killing, mice were perfused with 20 ml 0.1% Heparin-sulphate (Sigma Aldrich)-PBS through the heart and the caudal mesenteric arteries. Intestines were washed in PBS and were placed in 270 μl ice cold sample buffer (0.1 mg/ml STI, 0.05 m EDTA, 1 mM PEFABloc, 0.1% BSA, 0.05% Tween 20 in 1× PBS). In all, 30 μl of 20% saponin from quillaja bark (SigmaAldrich) were added into each tube and incubated overnight at 4 °C. Supernatants were collected following centrifugation at 14,000 *g* × 10 min at 4 °C and stored at −20 °C until further use.

### Measurement of antigen-specific antibody responses

Antibody titres were determined by MP305-specific ELISA. High-binding 96-well plates (Greiner BioOne) were coated with 50 μl per well of 5 µg/ml purified MP305 in 1× PBS and incubated overnight at room temperature (RT). The ELISA plates were washed three times with wash buffer (1× PBS/0.05% Tween 20 (Sigma Aldrich) (PBS-T)) and blocked with 0.1% BSA in 1× PBS for 30 min at 37 °C. The blocking solution was washed from the plates twice with 1× PBS. After washing, samples (fecal, tissue or serum) were added and serially diluted across the plate in 0.1% BSA/ PBS-T and incubated for 90 min at RT. Plates were washed once in 1× PBS. Horseradish peroxidase (HRP)-conjugated anti-mouse IgA (1:1500) (Southern Biotech) or HRP-conjugated anti-mouse IgG (1:4000) (Southern Biotech) were added to each well and incubated overnight at 4 °C. Plates were washed three times in wash buffer and one final time in 1× PBS. o-Phenylenediamine dihydrochloride substrate (1 mg/ml; Sigma Aldrich) was prepared in 0.1 m phosphate citrate buffer (pH 5) containing 4 μl H_2_O_2_ per 10 ml substrate and 50 μl were added per well. The plates were left to develop at RT and the reaction was then stopped by the addition of 25 μl/well of 1 m H_2_SO_4_, then the absorbance at 492 nm was read using a Microplate Reader (Thermo Scientific) running Scan-IT software (Thermo Scientific) to acquire data. Antibody concentrations were expressed as endpoint titres, which are the reciprocal of the highest analyte dilutions of fecal pellet supernatants or sera giving a reading above the cutoff plus two standard deviations. The cutoff was determined as the average of OD_492_ values of the control samples.

### IFNγ and IL-17A mRNA expression in stomach tissues

The samples preparation, RNA isolation, and real-time PCRs were performed as previously described.^19^

Primers used for real-time PCRs:

**Table:** 

IL-17A	Forward	5′-CCCTTGGCGCAAAAGTG-3′
	Reverse	5′-TCTTCATTGCGGTGGAGAGT-3′
IFNγ	Forward	5′-GCATAGATGTGGAAGAAAAGAGTCTCT-3′
	Reverse	5′-GGCTCTGCAGGATTTTCATGT-3′
β-actin	Forward	5′-CTGACAGGATGCAGAAGGAGATTACT-3′
	Reverse	5′-GCCACCGATCCACACAGAGT-3′

β-actin was used as the reference gene in all experiments. The difference between β-actin and the target gene (Δ*CT*) was determined, and the relative expression was calculated using the formula 2^Δ*CT*^. The values were adjusted so that the mean in the challenge control group was set to 1. The negative control (lacking reverse transcriptase) giving the lowest threshold cycle (*CT*) value was used to determine the detection limit.

### Statistics

The data represented in each graph are arithmetic means. For antibody studies, data were normalised with PBS control groups. One-way analysis of variance was used to determine significant differences between treatments and the degree of any significance was calculated by Dunetts and Tukey’s multiple comparison test. When required, a Mann–Whitney test was used to determine the degree of significance between two specific treatments. Prism5 (GraphPad) was used for all statistical analysis. *P* values < 0.05 were regarded as significant.

### Reporting summary

Further information on research design is available in the Nature Research Reporting Summary linked to this article.

## Supplementary information

Revised Supporting Information

Reporting summary

## Data Availability

All data generated or analysed during this study are included in this published article (and its supplementary information files). All relevant data are available upon request from the authors.
