# Supplementary material for: An oral alpha-galactosylceramide adjuvanted Helicobacter pylori vaccine induces protective IL-1R- and IL-17R-dependent Th1 responses
Source: NPJ Vaccines. 2019 Oct 25;4:45. doi: 10.1038/s41541-019-0139-z (PMC6814776; doi:10.1038/s41541-019-0139-z)
Supplement: Supplementary file 1 — Revised Supporting Information [file 41541_2019_139_MOESM1_ESM.pdf]

An oral alpha-galactosylceramide adjuvanted *Helicobacter pylori* vaccine induces protective IL-1R and IL-17R dependent Th1 responses

Running title: Alpha-Galactosylceramide adjuvanted *Helicobacter pylori* vaccine

Stephanie Longet[1¶], Aine Abautret-Daly[1¶], Christopher JH Davitt[1], Craig P McEntee[1], Vincenzo Aversa[2], Monica Rosa[2], Ivan S Coulter[2], Jan Holmgren[3], Sukanya Raghavan[3&*] and Ed C Lavelle[1,4&*].

[1]Adjuvant Research Group, School of Biochemistry and Immunology, Trinity Biomedical Sciences Institute, Trinity College Dublin, Dublin 2, D02 R590, Ireland.
[2]Sublimity Therapeutics Limited, Dublin City University, Alpha Innovation Campus, Old Finglas Road, Dublin, D11 KXN4, Ireland.
[3]University of Gothenburg Vaccine Research Institute, Department of Microbiology and Immunology, Institute of Biomedicine, University of Gothenburg, Box 435, 405 30 Gothenburg, Sweden.
[4]Centre for Research on Adaptive Nanostructures and Nanodevices & Advanced Materials Bio-Engineering Research Centre, Trinity College Dublin, Dublin 2, D02 PN40, Ireland.

***Corresponding authors:**
Prof Ed C. Lavelle
Adjuvant Research Group
School of Biochemistry and Immunology
Trinity Biomedical Sciences Institute
Trinity College Dublin,
D02 R590, Ireland.
Email: lavellee@tcd.ie
Phone: 353 1 8962488

Associate Prof Sukanya Raghavan
University of Gothenburg Vaccine Research Institute (GUVAX),
Dept. of Microbiology and Immunology,
Institute of Biomedicine
University of Gothenburg,
Box 435, 405 30 Gothenburg,
Sweden.
Email: sukanya.raghavan@microbio.gu.se

¶ these authors contributed equally
&*these authors jointly supervised the study

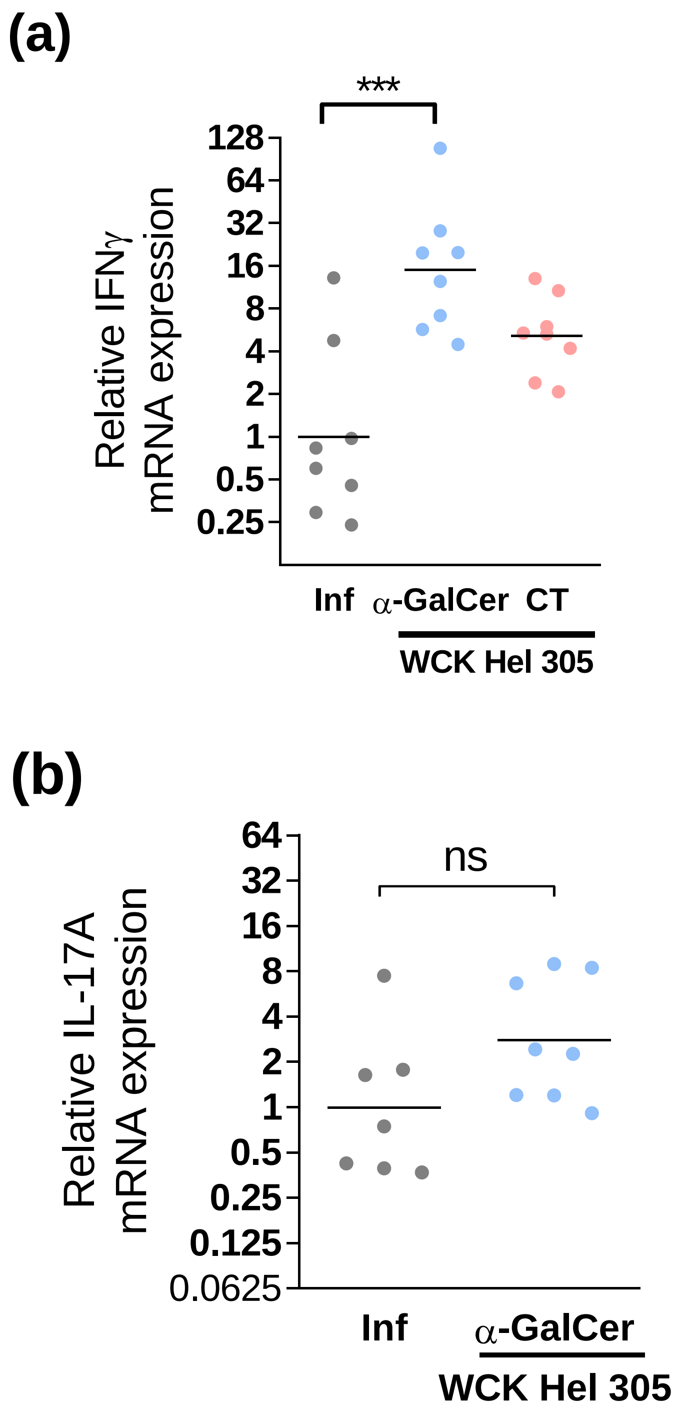

Supplementary Figure 1. Oral vaccination with α-GalCer adjuvanted Hel305 primes for an enhanced gastric IFN-γ response following infection.
WT mice were intragastrically immunised with whole-cell killed *H. pylori* Hel 305 adjuvanted with either α-GalCer or CT. Two weeks after the last round of immunisation, mice were challenged with live *H. pylori* SS1 bacteria. Three weeks post-challenge, stomach tissues were collected and RNA was extracted. Relative IFN-γ (**a**) or IL-17A (**b**) mRNA expression was analysed by RT-PCR. Unimmunised mice challenged at the same time-point served as infection controls (inf). Results present mRNA expression (geometric mean) for 7-8 mice per group. *** $p<0.001$.

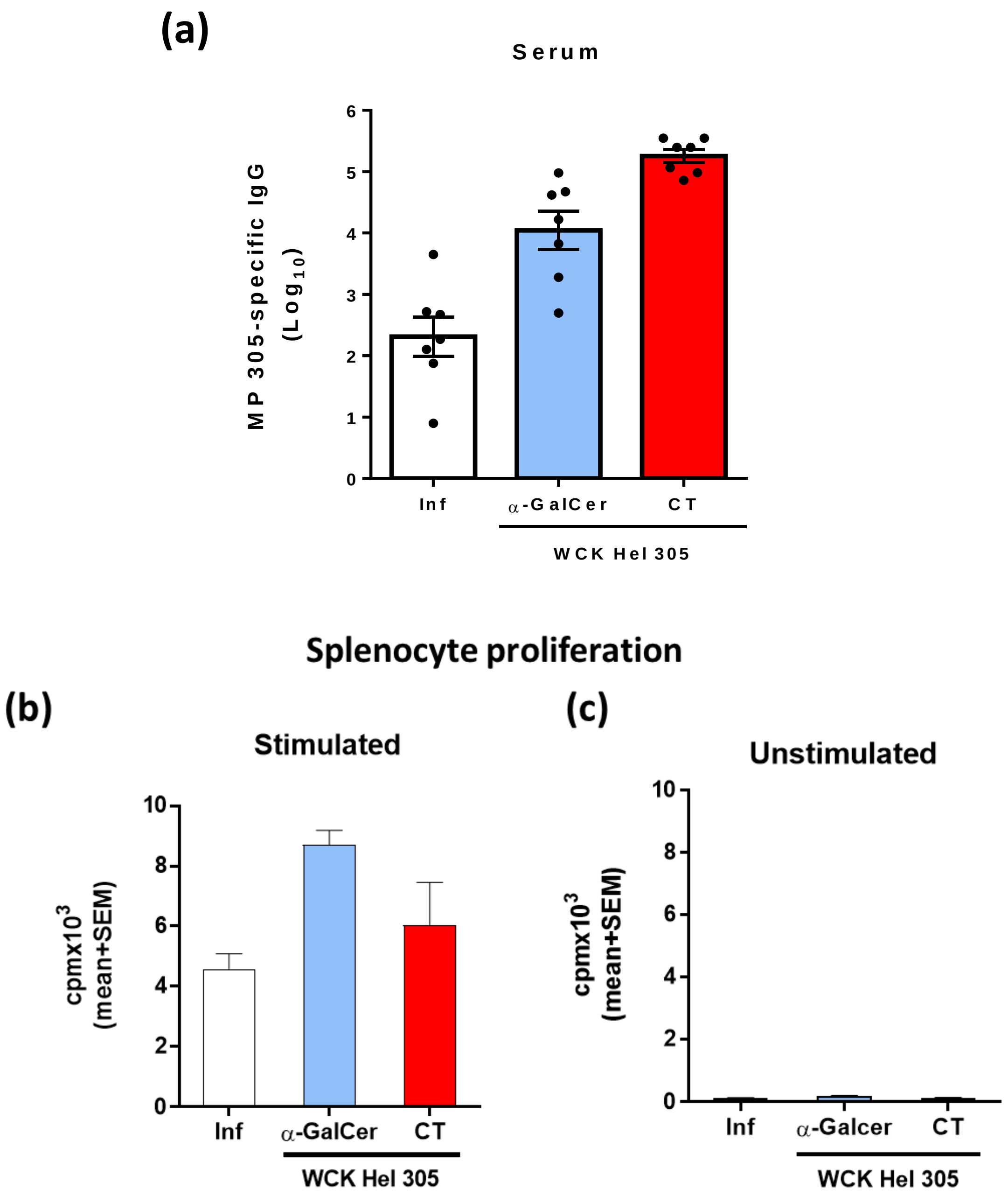

Supplementary Figure 2. Oral vaccination with α-GalCer adjuvanted Hel305 enhances antigen-specific systemic antibody and splenocyte proliferation.
WT mice were intragastrically immunised with whole-cell killed *H. pylori* Hel 305 with either α-GalCer or CT. Two weeks after the last round of immunisation, mice were challenged with live *H. pylori* SS1 bacteria. Three weeks post-challenge, sera and spleens were collected. MP305-IgG titers (**a**) were determined by ELISA and splenocyte proliferation (**b**) was analysed after restimulation *ex-vivo* with Hel 305 lysate antigen for 72 hours. As control experiment, splenocyte proliferation was analysed without restimulation (**c**). Unimmunised mice challenged at the same time-point served as infection controls (inf). Results present antibody titres (mean + SEM) (**a**) and splenocyte proliferation (cpm + SEM) (**b & c**) for 4-7 mice per group.

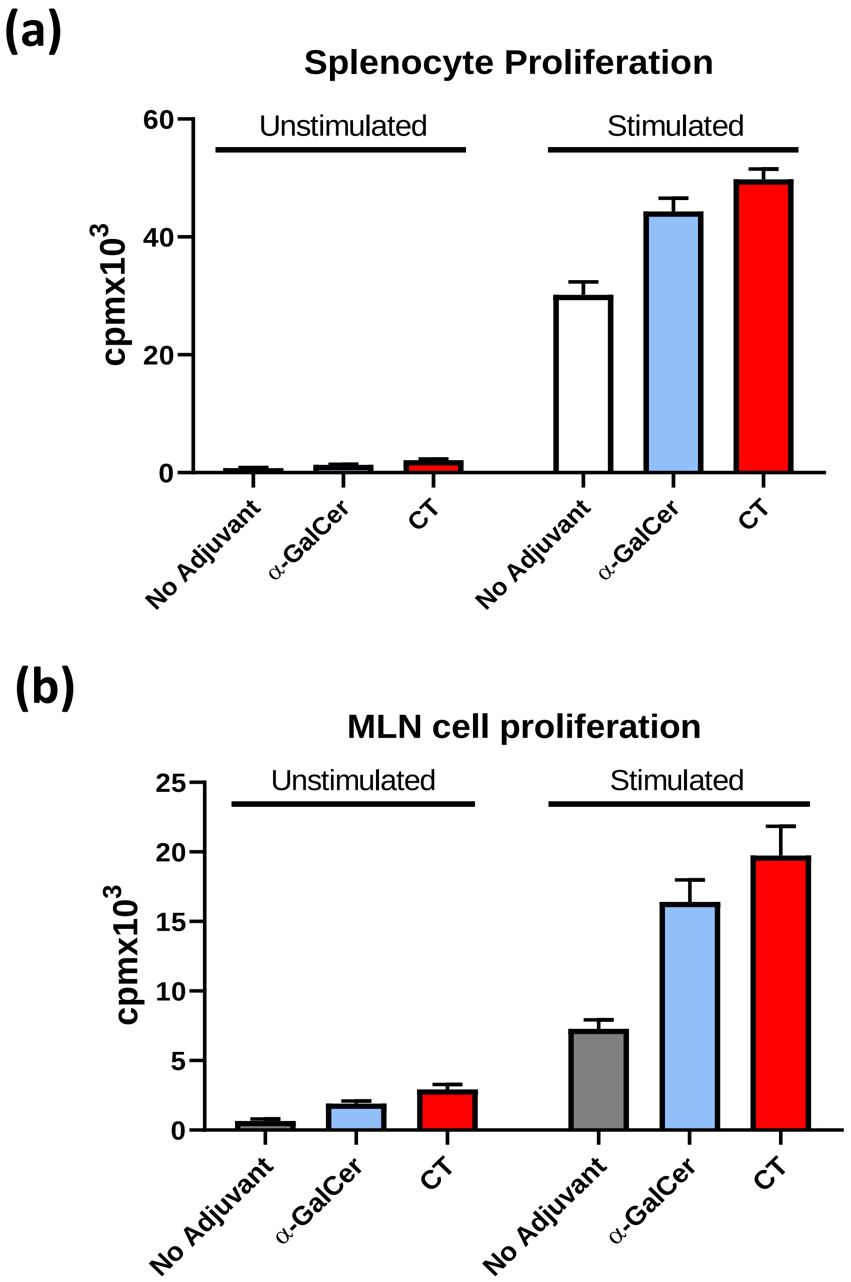

Supplementary Figure 3. Oral vaccination with Hel305 and α-GalCer promotes *H. pylori* antigen-specific splenocyte and MLN cell proliferation.
Mice were immunised intragastrically with whole-cell killed *H. pylori* Hel 305 with or without either α-GalCer or CT. Two weeks after the second round of immunisation, cells were isolated from the spleen (**a**) and mesenteric lymph nodes (**b**). Splenocyte and MLN cell proliferation were analysed after restimulation *ex-vivo* with purified MP305 lysate antigen for 72 hours or without restimulation. Results represent splenocyte and MLN cell proliferation (cpm (mean + SEM)) for 5 mice per group.

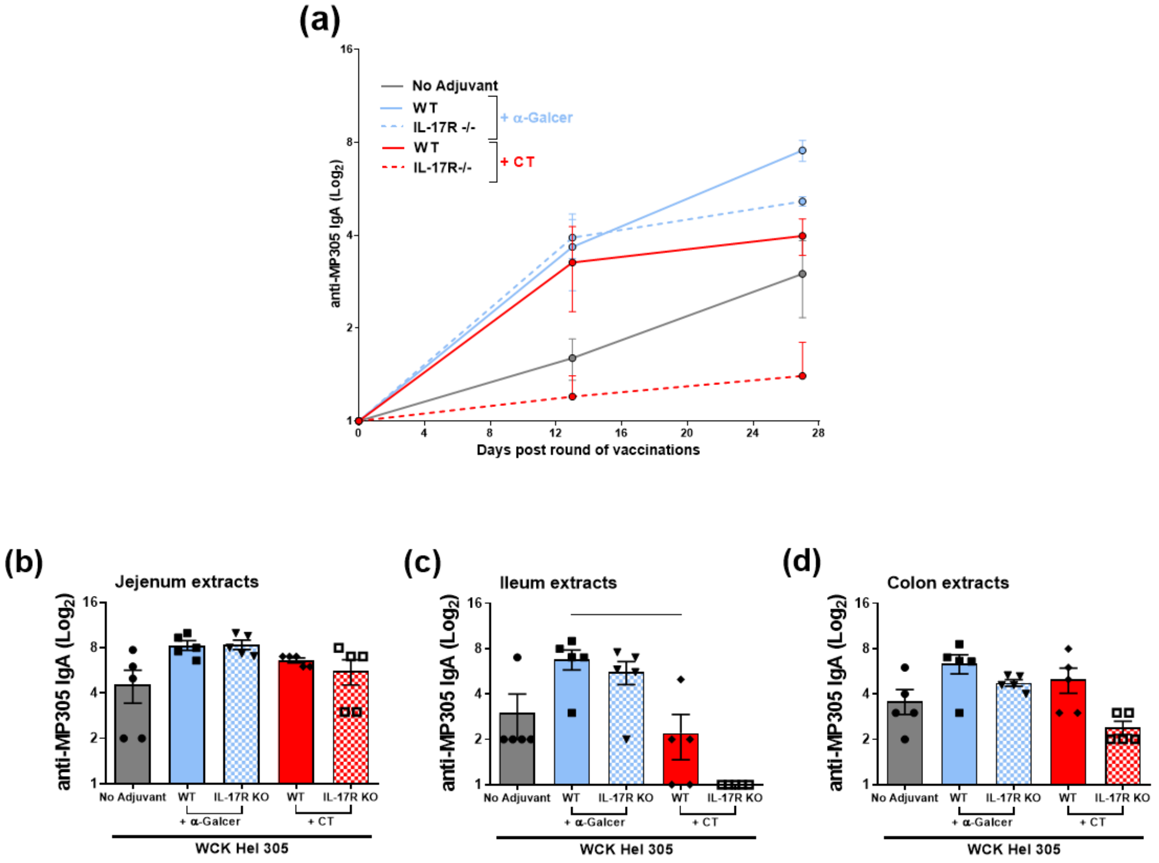

Supplementary Figure 4. Oral vaccination with α-GalCer adjuvanted Hel305 promotes IL-17 independent secretory IgA responses.

WT and IL-17R$^{-/-}$ mice were immunised with whole-cell killed *H. pylori* Hel 305 with or without either α-GalCer or CT on days 0 and 1 then, on days 14 and 15. MP305-specific IgA titres in faecal pellet supernatants were determined by ELISA before each round of immunisation and two weeks after the last round of immunisation. The graph line shows the evolution of IgA titres post-immunisation for each immunised group of mice (**a**). Two weeks after the last round of immunisation, mice were perfused to remove the blood from the organs and tissues were collected. *H. pylori* MP305-specific IgA titres were measured in jejunum (**b**), ileum (**c**) and colon (**d**) extracts. Results present antibody titres (mean + SEM) for 5 mice per group. Data representative of 3 independent experiments with n=5 mice/group and experiment. ** p<0.01, *** p<0.001.

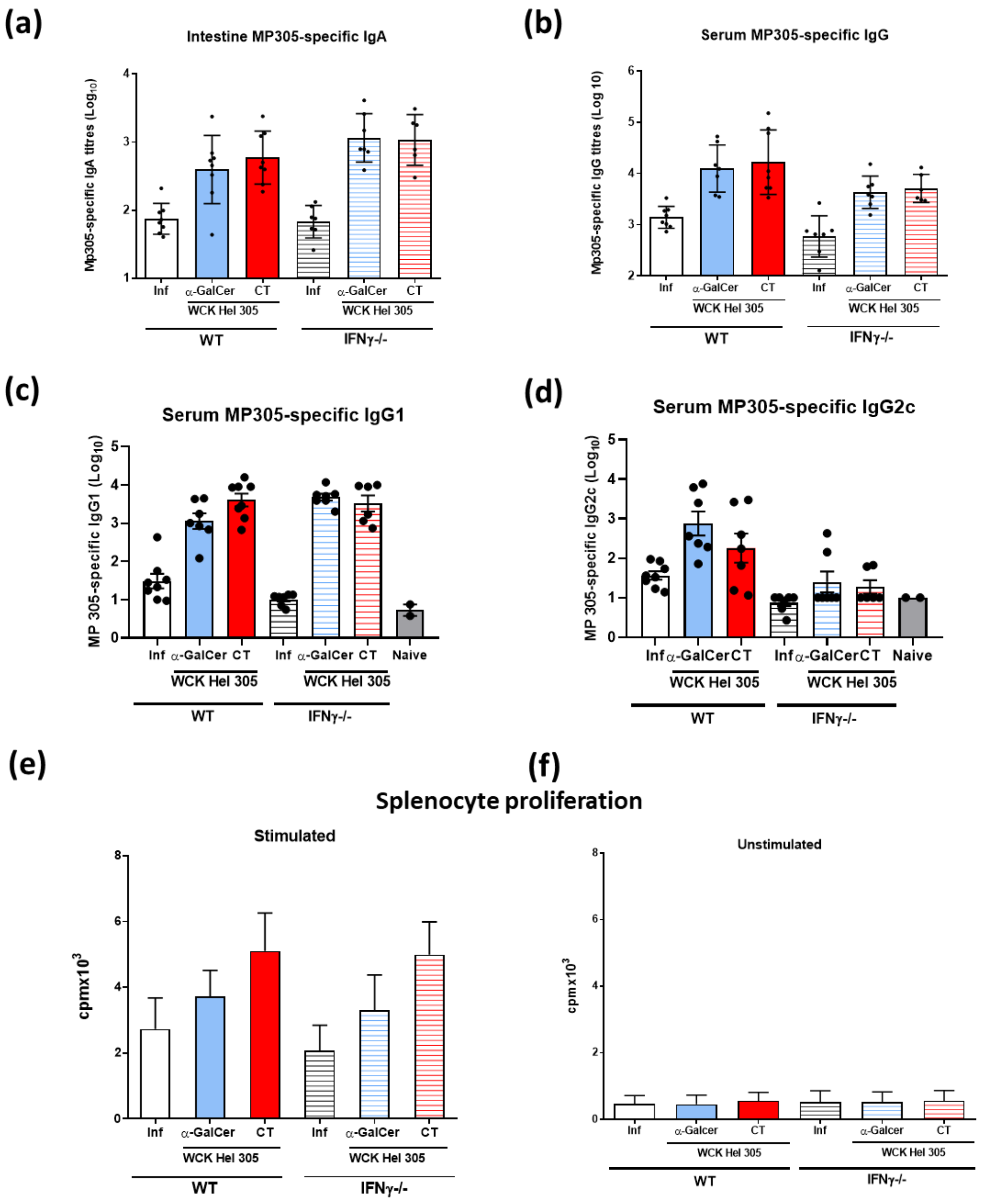

Supplementary Figure 5. Oral vaccination with α-GalCer adjuvanted Hel305 enhances antigen-specific faecal IgA, serum IgG and IgG1 responses and splenocyte proliferation in an IFNγ independent manner.

WT and IFNγ$^{-/-}$ mice were intragastrically immunised with whole-cell killed *H. pylori* Hel 305 with either α-GalCer or CT and two weeks after the last round of immunisation, mice were challenged with live *H. pylori* SS1 bacteria. Three weeks after the challenge, sera and spleens were collected. Intestinal MP305-IgA (**a**) as well as serum IgG (**b**), IgG1 (**c**) and IgG2c (**d**) titres were determined by ELISA. Splenocyte proliferation were analysed after restimulation *ex-vivo* with purified MP305 lysate antigen for 72 hours (**e**) or without restimulation (**f**). Unimmunised mice challenged at the same time-point served as infection controls (inf). Results represent antibody titres (mean + SEM) (**a, b, c & d**) and splenocyte proliferation (cpm (mean + SEM)) (**e & f**) for 7-8 mice per group.

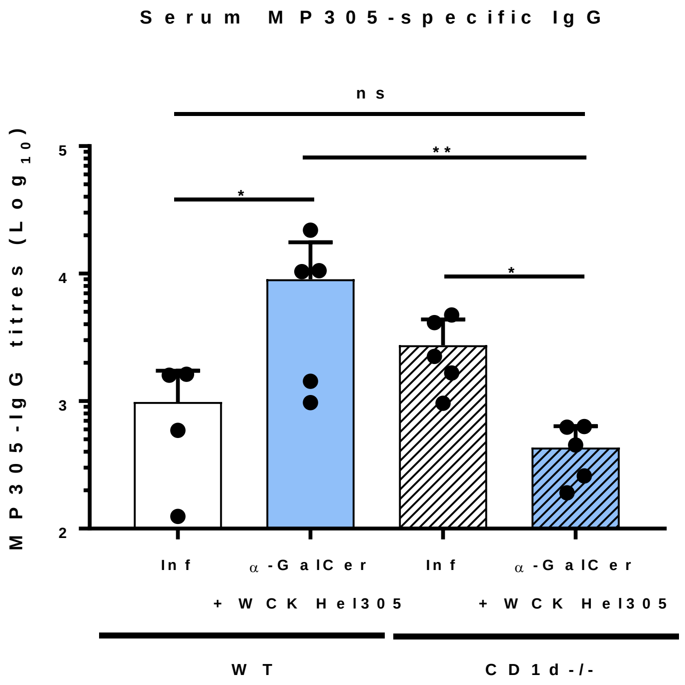

Supplementary Figure 6. Antigen-specific IgG responses induced by oral vaccination with whole-cell killed *H. pylori* adjuvanted with α-GalCer requires the antigen-presenting protein CD1d.

WT and CD1d$^{-/-}$ mice were immunised intragastrically with whole-cell killed *H. pylori* Hel 305 with α-GalCer. Two weeks after the last round of immunisation, mice were challenged with live *H. pylori* SS1 bacteria. Three weeks post-challenge, serum was collected and MP305-IgG titres were determined by ELISA. Unimmunised mice challenged at the same time-point served as infection controls (inf). Data represent geometric means + SEM for 4-5 mice per group. * p<0.05, ** p<0.01.
